# Association between interindividual variability in training volume and strength gain

**DOI:** 10.3389/fphys.2022.983478

**Published:** 2022-09-26

**Authors:** Ryoichi Ema, Itaru Saito, Ryota Akagi

**Affiliations:** ^1^ Faculty of Sport Science, Shizuoka Sangyo University Owara, Iwata-shi, Japan; ^2^ College of Systems Engineering and Science, Shibaura Institute of Technology Fukasaku, Minuma-ku, Saitama, Japan; ^3^ Graduate School of Engineering and Science, Shibaura Institute of Technology Fukasaku, Minuma-ku, Saitama, Japan

**Keywords:** high responders, hip flexion, isometric training, knee extension, low responders, maximal voluntary contraction

## Abstract

This study aimed to examine the association between interindividual variability in strength changes and in training volume. A total of 26 untrained men completed 4-weeks of isometric knee extension (KE group, *n* = 12) and hip flexion (HF group, *n* = 14) training. Each training session comprised four sets of ten isometric contractions, 3-s contractions every 20 s. Training volume, which was defined as impulse during contractions, and maximal voluntary contraction (MVC) torque during KE and HF were evaluated. Based on the magnitude of MVC torque changes, the participants were divided into the high and low responders (*n* = 13; KE = 6 and HF = 7 per responders). The MVC torque changes (KE, 20.8%; HF, 22.4%) and total training volume did not significantly differ between the two groups. A higher training volume was demonstrated in the low responders than the high responders. The total training volume was positively associated with the MVC torque changes in low responders (r = 0.869%, 95% confidence interval [0.610, 0.960], *p* < 0.001), but not in high responders [r = 0.229, 95% confidence interval (−0.368, 0.693), *p* = 0.451], KE or HF group. Results showed that training volume was an important factor in determining the magnitude of strength gains in low responders, and MVC torque could improve by approximately 20% with the use of the study protocol regardless of joint actions involved during training.

## Introduction

High-intensity isometric training stimulates greater strength improvements than low-intensity isometric training (Szeto et al., 1989). Additionally, a systematic review (Oranchuk et al., 2019) has shown that the total training volume is more important than training intensity to gain strength improvement. In previous studies, training volume, such as percentage of maximal voluntary contraction (MVC) × duration of contraction per set × number of sets per session (Kanehisa et al., 2002) and/or total impulse (areas under the force-time training curve) (Young et al., 1985), were controlled using different training intensity protocols. For example, a group showed a 60.3% increase in MVC torque induced by isometric training at 100% of MVC (6 s × 12 sets), whereas another group demonstrated a 61.0% increase at 60% of MVC (30 s × 4 sets) (Kanehisa et al., 2002). In addition, Tillin and Folland (2014) compared two types of isometric training (force exertion over 1 s, up to 75% of MVC, hold for 3 s vs. contraction as fast and hard as possible up to 90% of MVC for −1 s). They found that the former stimulated a greater improvement in MVC strength than the latter. These studies compared strength changes between groups (e.g., high intensity vs. low intensity) or limbs (e.g., right leg vs. left leg) in a participant, and a group average was considered as a typical response in most individuals. However, the interindividual variability in training volume was not evaluated. From the point of view of the mechanical stress during training with 100% MVC intensity, the actual training volume can be different among the participants owing to variabilities in time-course changes in strength during a training period (Komi et al., 1978) and to the ability of maintaining MVC during contractions [e.g., fatigue resistance (Miyamoto et al., 2013)]. Thus, when considering the individual training volume in isometric training with maximal effort, it should be defined using impulse rather than other parameters. Variability in training effects may be associated with variability in training volume. Furthermore, the concept of high versus low responders may provide insights about the mechanisms of training adaptation (Mann et al., 2014).

The applicability of a training protocol is important when expecting training responses in the field of training and/or rehabilitation. To organize training programs according to evidence, whether the effect of a training protocol on a joint is applicable in the training of other joints must be validated. If some studies that investigated strength changes by isometric training at the same intensity but different training protocols (e.g., number of contractions, sets, durations) and target muscles are picked up, the changes per week were not consistent among the previous studies (Oranchuk et al., 2019). These may suggest that a training protocol reported in a previous study does not guarantee the training adaptations when the protocol is applied to other muscle groups. To the best of our knowledge, previous studies have not evaluated training effects between different target muscles with similar training protocols and the associations between training effects and volume.

This study aimed to investigate the relationship between interindividual variability in strength changes and in training volume. Moreover, whether similar strength gains are obtained by training for different joint actions after controlling these protocols was evaluated. This research is part of a large study, and some data have been reported in a previous study (Ema et al., 2018) with different purpose and conclusion.

## Materials and methods

### Participants

This study was approved by the Ethics Committee of the Shibaura Institute of Technology (#16-009) and conducted according to the Declaration of Helsinki. The participants were informed about the purpose and potential risks of the study, and a written informed consent was obtained. A total of 26 untrained healthy young men completed a 4-weeks isometric training. The inclusion criteria were that the participants did not participate in any resistance training activities of the lower extremity for at least 2 years and did not have any previous or current knee and hip injuries. Daily physical activity prior to the training was evaluated using a previously validated questionnaire (Craig et al., 2003).

### Procedures

The participants attended a familiarization about the study 3–7 days before the baseline (PRE) measurement. Five to 7 days after PRE, the participants completed 4-weeks of isometric training. Two kinds of training that involve different joint actions after controlling for protocols were performed to identify whether the changes in MVC strengths were similar between muscle groups. Two to 4 days after the last training session, post-training (POST) measurement was conducted. To examine the relationship between the training volume and strength changes, data about torque were recorded during the training periods at 1 kHz and PRE and POST measurements at 4 kHz using an A/D converter (PowereLab16/35, ADInstruments, Australia).

**FIGURE 1:**
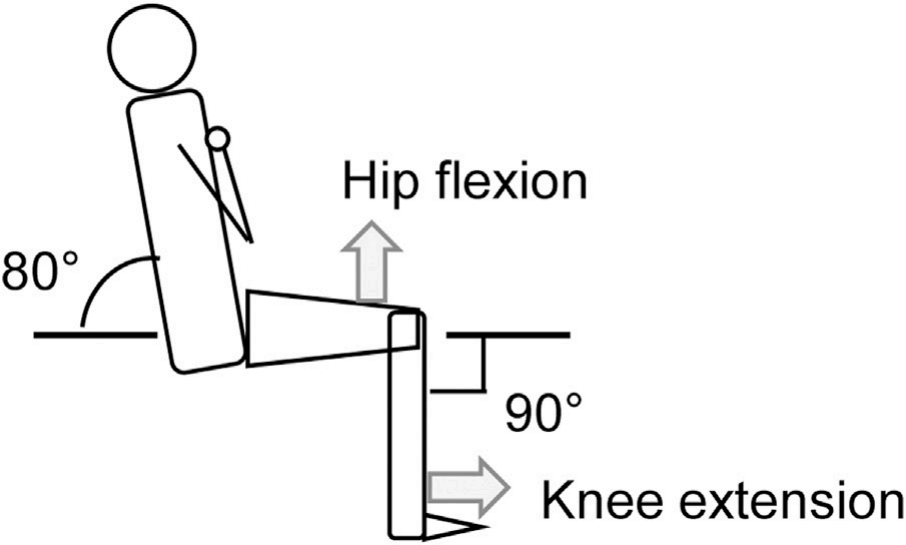
Schematic illustration of the posture for the maximal voluntary contraction torque measurements and isometric training.

The participants performed isometric unilateral (right leg) KE training (KE group, *n* = 12) or hip flexion (HF) training (HF group, *n* = 14) 3 times per week for 4 weeks using a dynamometer (VTK-002, VINE, Japan) at hip and knee joint angles of 80° and 90° (full extension = 0°), respectively (Figure 1). They were assigned to one of the training groups based on age, height, and body mass (mean ± standard deviation [SD]: KE group, 22 ± 3 years, 170 ± 4 cm, 61 ± 7 kg; HF group, 22 ± 2 years, 169 ± 3 cm, 61 ± 8 kg). Each training session comprised 4 sets of 10 isometric contractions, 3-s contractions every 20 s. A 2-min rest was provided between sets. Maximum force was exerted as fast and hard as possible. The participants received verbal encouragement during contractions. PRE and POST measurements were obtained with a dynamometer (CON-TREX MJ, PHYSIOMED, Germany).

In each training session, total impulse, areas under the time-torque curve of 40 contractions, were defined as the training volume for each session. The training volume of each session (2nd to 12th session) was defined as the ratio of the first session, and total volume was calculated. Therefore, if a participant produced the same impulse during each session in the training period, the total volume corresponded to 12.

At PRE and POST measurements, after conducting the warm-up procedures consisting of submaximal contractions at intensities of 30%, 50%, and 80% of MVCs, the participants performed KE and HF twice with maximal effort. The measurement posture was the same to that during the training (Figure 1). MVC torque was defined as the peak torque of each contraction. A 1-min rest was provided between contractions. MVC torque was normalized to body mass. The means of the two contractions were used for later analyses.

### Statistical analyses

Data are presented as mean ± SD. All analyses were conducted with SPSS version 25 (IBM Inc., United States). The normality of the data was checked by the Shapiro-Wilk test, and physical activity values were not normally distributed. Thus, the data were log-transformed before analyses. In addition to comparing the KE and HF groups, subgroup analyses were conducted. The participants in each group were further divided into the high (*n* = 13; KE = 6 and HF = 7) and low responder (*n* = 13; KE = 6 and HF = 7) in descending order based on the magnitude of MVC torque changes. The participants in the current study were untrained young men, and the training protocol was controlled between the KE and HF groups. Thus, it would be difficult to consider that the relationships between training volume and strength changes are substantially different between these groups, and high and low responders were comprised of both groups. A two-way (group [KE, HF] × time [PRE, POST] or group [KE and HF, high and low] × session) analysis of variance (ANOVA) was used for dependent variables. The between-group/responder differences in dependent variables were investigated using the independent *t*-test. When a significant main effect and/or interaction was evident, Bonferroni multiple comparisons were conducted. The Pearson’s product-moment correlation coefficients (r) and partial correlation coefficients adjusted for baseline MVC torque were used to examine the relationships between variables. A *p* < 0.05 was considered statistically significant. The smallest practically important correlation of r was 0.1 (Hopkins et al., 2009). The 95% confidence interval (CI) of r was determined. If the limit was > |0.1| with a P of <0.05, the relationship was assumed to be substantial. CV was calculated if appropriate.

## Results

A significant main effect of time (*p* < 0.001) without interaction of group × time demonstrated that MVC torque significantly increased in both groups. The dependent variables did not significantly differ between the KE and HF groups (Table 1). There were no significant differences in terms of age, height, and body mass between the high and low responders. Meanwhile, the baseline MVC torque and total training volume in the low responders were significantly higher than those in the high responders.

**TABLE 1:** Dependent variables in each group and in each responder.

		KE group (*n* = 12)			CV (%)	HF group (*n* = 14)			CV (%)	*p* Value
Physical activity	MET min/wk	2,534	±	2,233	88.1	2,443	±	1703	69.7	0.923
MVC torque, before	N·m·kg^−1^	2.60	±	0.36	14.0	2.52	±	0.39	15.6	0.591
MVC torque, after	N·m·kg^−1^	3.08	±	0.38	12.4	3.03	±	0.38	12.6	0.723
Total training volume	a.u.	13.6	±	1.3	9.2	13.4	±	1.6	11.9	0.657
Changes in MVC torque	%	20.8	±	9.9	47.3	22.4	±	12.8	57.2	0.737
		high responders (*n* = 13)			CV (%)	Low responders (*n* = 13)			CV (%)	*p* Value
Physical activity	MET min/wk	1984	±	1,174	59.2	2,986	±	2,407	80.6	0.492
MVC torque, before	N·m·kg^−1^	2.36	±	0.30	12.9	2.75	±	0.34	12.5	0.006
MVC torque, after	N·m·kg^−1^	3.04	±	0.34	11.1	3.07	±	0.42	13.8	0.851
Total training volume	a.u.	12.8	±	1.2	9.5	14.2	±	1.3	9.2	0.010
Changes in MVC torque	%	31.0	±	6.7	21.7	12.4	±	5.9	47.8	<0.001

Data are shown as mean ± standard deviation. CV, coefficient of variation; HF, hip flexion; KE, knee extension; MET, metabolic equivalent; MVC, maximal voluntary contraction.

**FIGURE 2:**
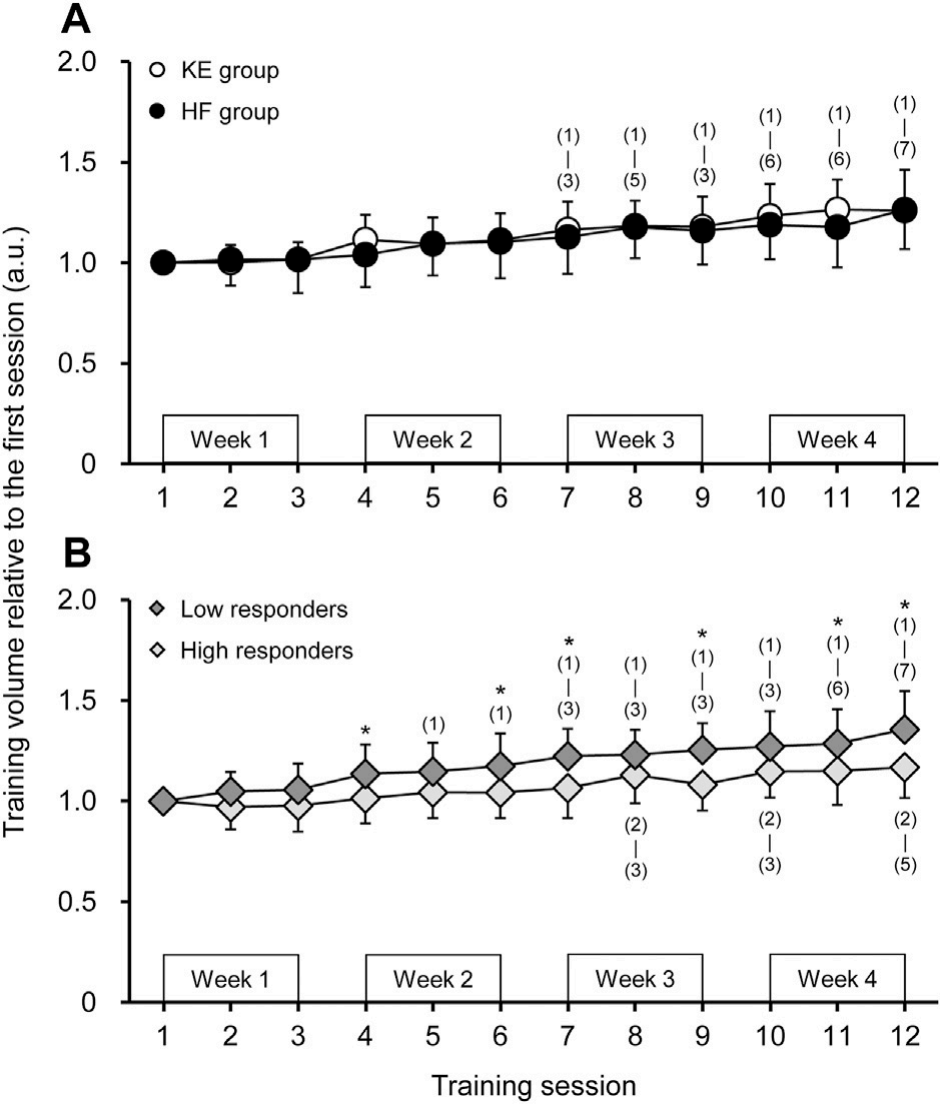
Training volume in each training session in **(A)** the knee extension (KE) and hip flexion (HF) groups and in **(B)** the high and low responders. The number inside the parenthesis indicates the training session, where a significant difference was found. *, a significant difference was observed between the high and low responders.

No significant interaction of group × session with a main effect of session (*p* < 0.001) was observed for training volume (Figure 2). The time-course changes in training volume did not significantly differ between the KE and HF groups. In contrast, there was a significant group × session interaction (*p* = 0.034) for the comparisons of high and low responders. The low responders had a faster and higher increase in training volume than the high responders.

**FIGURE 3:**
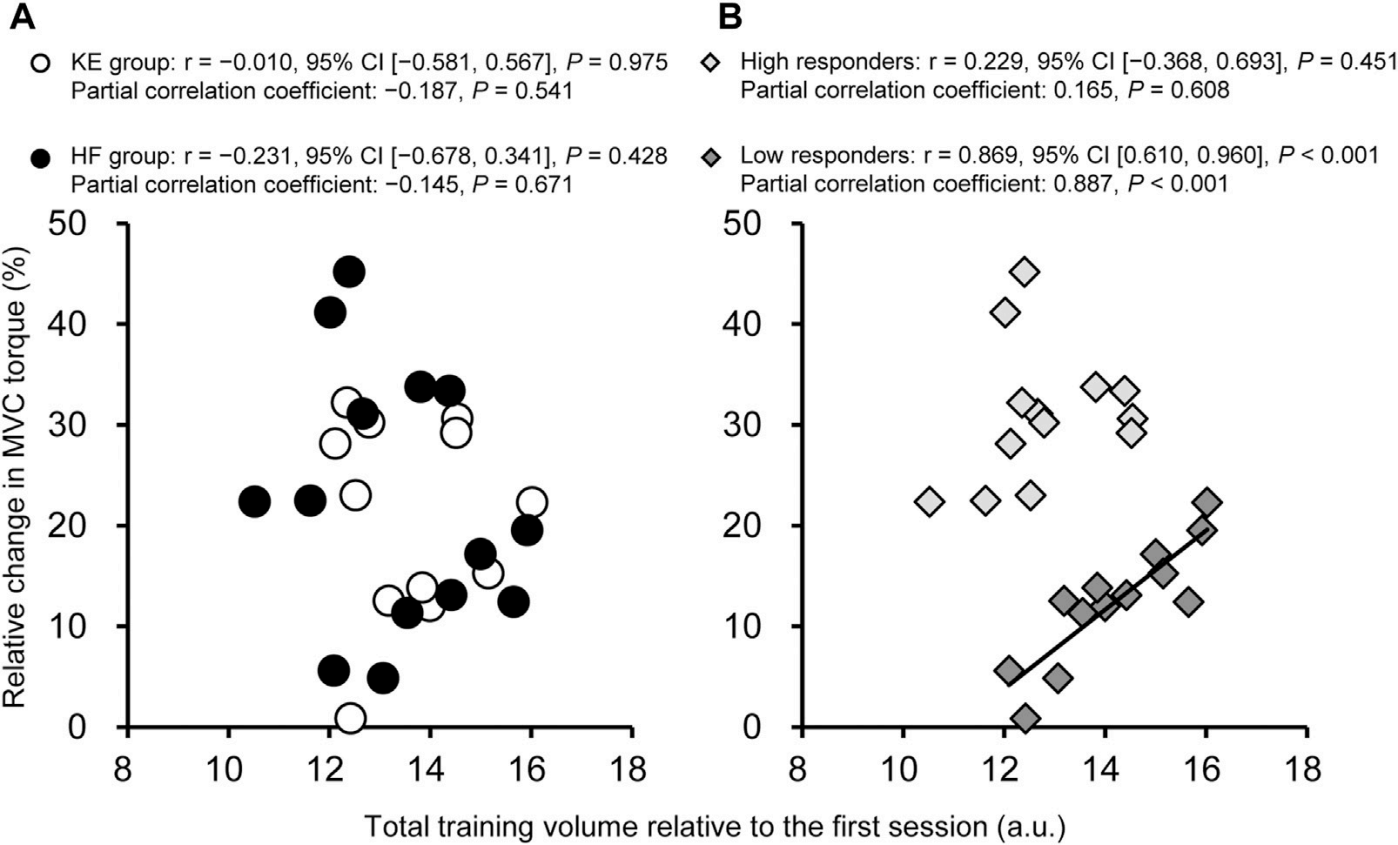
The relationships between the total training volume and changes in maximal voluntary contraction (MVC) torque in **(A)** the knee extension (KE) and hip flexion (HF) groups and in **(B)** the high and low responders. Changes in MVC torque were determined based on the KE MVC torque values of the KE group and the HF MVC torque values of the HF group. CI, confidence interval.

There was no significant correlation between the total training volume and relative MVC torque changes in the KE and HF groups (Figure 3). In contrast, the total volume was positively correlated with MVC torque changes in the low responders, but not in the high responders. The relationships did not change after controlling for baseline MVC torque using the partial correlation coefficients. A significant positive correlation was observed between the training volume at week 2 (r = 0.835, 95% CI [0.526, 0.949], *p* < 0.001), week 3 (r = 0.843, 95% CI [0.545, 0.952], *p* < 0.001), and week 4 (r = 0.870, 95% CI [0.613, 0.961], *p* < 0.001) and MVC torque changes in the low responders and between the training volume at week 1 (r = 0.657, 95% CI [0.166, 0.9887], *p* = 0.015) and MVC torque changes in the high responders. A significant negative correlation was observed between baseline strength and strength gains only in the HF group (r = −0.555%, 95% CI [−0.839, −0.035], *p* = 0.039). However, the significance did not reach the substantial level.

## Discussion

Only the low responders showed a distinct relationship between the total training volume and the magnitude of strength improvement. This was not a spurious relationship owing to variabilities in baseline strength. A higher training volume was demonstrated in the low responders than the high responders. Therefore, training volume could not likely determine whether strength gain is above/below average in a certain training group; however, it may determine the individual adaptation when low responders were selected.

The lack of significant relationship between the training volume and MVC torque changes in the high responders could be partly attributed to the relatively low variability in strength changes in the high responders (CV = 21.7%) than the low responders (CV = 47.8%, Table 1). This finding is caused by variability in parameters affecting the magnitude of correlation (Goodwin and Leech, 2006). However, two participants had gains of >40%, and this can more likely explain the discrepancy observed between the responders. Without these participants, the Pearson’s product-moment correlation coefficient (r = 0.692%, 95% CI [0.158, 0.913], *p* = 0.018) and partial correlation coefficient adjusted for baseline strength (0.700, *p* = 0.024) in the high responders will show substantial levels.

The baseline MVC torque relative to body mass was significantly lower in the high responders than in the low responders, and the corresponding difference was not noted after the intervention (Table 1). Both responders showed no significant relationships between the baseline strength and strength changes. These findings may suggest that although baseline strength determines whether the training response of a participant is above/below average in a certain training group, it does not relate to the individual variability in strength gains. In a previous study, the magnitude of neural activation at the baseline was associated with changes in MVC strength (Gondin et al., 2005). Thus, neural activation at the baseline might have been lower in the high responders than in the low responders, which led to lower baseline strength and greater strength gains in the high responders. However, greater training volume may not be related to greater neural adaptations, because training volume was greater in the lower responders than in the high responders (Table 1).

The magnitude of changes in knee extensor MVC torque in the KE group (20.8% ± 9.9%) and hip flexor MVC torque in the HF group (22.4% ± 12.8%) did not significantly differ. Such mean values and variabilities were similar to those obtained in the previous study (Tillin and Folland, 2014), which observed a 21% ± 12% increase in MVC strength after isometric training for 4 weeks. Moreover, the participants exerted force over a 1-s period, up to 75% of MVC, hold for 3 s (Tillin and Folland, 2014). The number of sets and contractions was similar to that of the present study. Thus, training protocols such as isometric training at high intensity, four sets of ten contractions, 3 s for each contraction, for 4 weeks might improve MVC strength by approximately 20% on average.

The present study had several limitations. First, it included a relatively small sample. Hence, we identified a substantial level of r using not only *p* value but also 95% CI. It is noted that the range of CI is affected by the number of samples. The lower limit for the relationships between training volume and MVC torque changes in the low responders was above the large (0.5) effect size (Hopkins et al., 2009). Thus, the substantial correlations observed in the current study may provide good evidence, which can be used as a basis for practical applications in training and coaching. However, further research with a large sample is required. Second, the current study did not report changes in neural activations and muscle sizes, and the participants were limited to untrained men. To highlight the current findings, it is necessary to examine whether similar results are obtained with more physiological data and for different populations (e.g., women, trained participants, and older adults). Finally, the effect of random error in strength testing should be considered. The mean CVs in the time-matched control group (Ema et al., 2018) were 1.8% and 3.6% for knee extensor and hip flexor MVC torque, respectively. The differences in the magnitude of MVC torque changes between the bottom of the high responders and top of the low responders were 0.7% in the KE group and 2.8% in the HF group, respectively. Thus, the order of bottom of the high responders or top of the low responders in the KE and HF group may be related to the random error. Thus, we additionally investigated the relationships between the training volume and strength changes after excluding four participants (*n* = 11 in each responder). However, the main findings did not change (high responders: r = −0.046, *p* = 0.892; low responders: r = 0.790, *p* = 0.004), thereby indicating that random error might not have affected the current findings.

There are some practical applications. The current study demonstrated that training volume would be an important factor in determining the magnitude of strength gains in low responders. The low responders indicated higher baseline strength relative to body mass than the high responders. Thus, the findings may apply to athletes considering that they have relatively high strength-to-weight ratio compared with control participants (Hori et al., 2021). In addition, the MVC torque changes were correlated to the training volume during week 2 and thereafter. Considering these, coaches/athletes could assess training responses by investigating the strength-to-weight ratio at the baseline and the increase in training volume from the first session in the middle of training. Moreover, based on the results of the current study, coaches/athletes may organize training programs that aim to improve the performance of a joint.

In conclusion, the present study demonstrated the association between total training volume and magnitude of strength gains in the low responders. They showed higher MVC torque relative to body mass at baseline and training volume than the high responders. These suggest that training volume is not likely determine whether strength gain is above/below average in a certain training group, but it may determine the individual adaptation when participants who have a relatively high strength-to-weight ratio before intervention were selected. The MVC strength can improve by approximately 20% with the use of the current isometric resistance training regardless of joint actions involved during training.

## Data Availability

The raw data supporting the conclusion of this article will be made available by the authors, without undue reservation.
